# P-490. Differentiation of Latent Versus Active HIV Infection at the Single Cell Level Using RT-ddPCR

**DOI:** 10.1093/ofid/ofae631.689

**Published:** 2025-01-29

**Authors:** Olivier Pernet, Qinlan Wu, Patricia anthony, Gwyndolyn McCaney, Andrea Kovacs

**Affiliations:** University of Southern California, Los Angeles, California; University of Southern California, Los Angeles, California; USC, Los Angeles, California; USC, Los Angeles, California; Keck School of Medicine, University of Southern California, Los Angeles, CA

## Abstract

**Background:**

With successful treatment of HIV, viral loads can drop to levels undetectable by regular qPCR diagnostic methods. However, the virus remains latent in cellular reservoirs . These reservoir cells are rare, and their quantification is a critical parameter to assess future HIV treatments. Digital PCR (dPCR) technology bypasses the limitation inherent to regular qPCR, and can quantify to some extent cells with latent HIV infection within a cell population.

**Methods:**

Here we describe a protocol relying on dPCR that allows differentiation between active and latent infection at the single cell level. We used a digital droplet setting (ddPCR) that allows for reverse transcription (RT) within the droplet. The assay is multiplexed to detect both HIV proviral DNA as well as spliced mRNA indicative of HIV active replication. Briefly, single cell are isolated into droplet containing RT-ddPCR reaction, including the Bio-Rad Supermix One-Step RT ddPCR Advanced Kit and primers/probe sets targeting HIV genome *gag* in the FAMlow channel and the spliced 2kb class mRNA in the FAMhigh channel. After the RT-PCR reaction, the droplet fluorescence intensity was measured using a Bio-Rad Q200 droplet reader.

**Results:**

We successfully evaluated HIV activation in cells lines with (8E5) or without HIV (CEM-T4) infection, as well as volunteer Peripheral Blood Mononuclear Cells (PBMC). We first determine the fluorescence bandwith for each target using DNA/RNA purified from cell lysate, identifying the HIV genomic DNA signal (FAMlow) between 2500 and 3500 MFI and the active replication signal (FAMhigh) between 4000 and 5000 MFI. We then repeated the RT-ddPCR reaction, this time using cells isolated in an excess of droplets (2000 cells for 15,000 droplet per reaction).

**Conclusion:**

This

Single Cell RT-ddPCR protocol allows for identification and quantification of HIV infected cells and assess their individual level of activation/latency. In the future, the multiplexing approach could be used to not only assess HIV activation, but also to identify cellular transcripts specific to latency or active replication.

**Disclosures:**

**All Authors**: No reported disclosures

